# Vaginal Microbiota and Cytokine Levels Predict Preterm Delivery in Asian Women

**DOI:** 10.3389/fcimb.2021.639665

**Published:** 2021-03-04

**Authors:** Manoj Kumar, Selvasankar Murugesan, Parul Singh, Marwa Saadaoui, Duaa Ahmed Elhag, Annalisa Terranegra, Basirudeen Syed Ahamed Kabeer, Alexandra K. Marr, Tomoshige Kino, Tobias Brummaier, Rose McGready, François Nosten, Damien Chaussabel, Souhaila Al Khodor

**Affiliations:** ^1^Research Department, Sidra Medicine, Doha, Qatar; ^2^Shoklo Malaria Research Unit, Mahidol-Oxford Tropical Medicine Research Unit, Faculty of Tropical Medicine, Mahidol University, Mae Sot, Thailand; ^3^Centre for Tropical Medicine and Global Health, Nuffield Department of Medicine, University of Oxford, Oxford, United Kingdom; ^4^Swiss Tropical and Public Health Institute, Basel, Switzerland; ^5^University of Basel, Basel, Switzerland

**Keywords:** microbiota, microbiome, 16S rRNA gene sequencing, dysbiosis, vaginal cytokines, Nugent scoring, Asian, Preterm birth

## Abstract

Preterm birth (PTB) is the most common cause of neonatal morbidity and mortality worldwide. Approximately half of PTBs is linked with microbial etiologies, including pathologic changes to the vaginal microbiota, which vary according to ethnicity. Globally more than 50% of PTBs occur in Asia, but studies of the vaginal microbiome and its association with pregnancy outcomes in Asian women are lacking. This study aimed to longitudinally analyzed the vaginal microbiome and cytokine environment of 18 Karen and Burman pregnant women who delivered preterm and 36 matched controls delivering at full term. Using 16S ribosomal RNA gene sequencing we identified a predictive vaginal microbiota signature for PTB that was detectable as early as the first trimester of pregnancy, characterized by higher levels of *Prevotella buccalis*, and lower levels of *Lactobacillus crispatus and Finegoldia*, accompanied by decreased levels of cytokines including IFNγ, IL-4, and TNFα. Differences in the vaginal microbial diversity and local vaginal immune environment were associated with greater risk of preterm birth. Our findings highlight new opportunities to predict PTB in Asian women in low-resource settings who are at highest risk of adverse outcomes from unexpected PTB, as well as in Burman/Karen ethnic minority groups in high-resource regions.

**Graphical Abstract:**
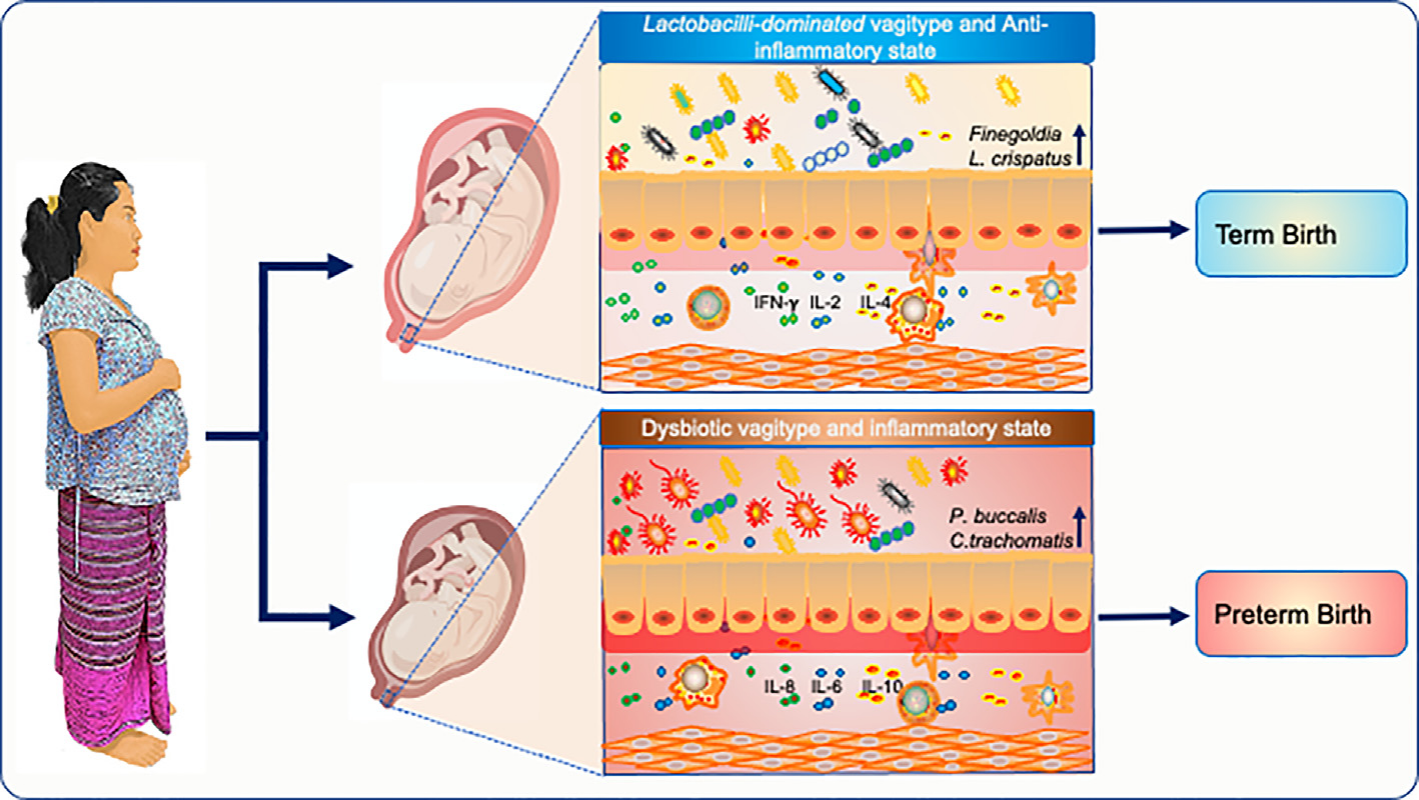


## Introduction

Preterm birth (PTB), defined as birth before 37 weeks of gestation, is the leading worldwide cause of neonatal mortality, and of morbidity and mortality in children under five in high income countries (Blencowe et al., 2012). Nearly 15 million pregnant women experience PTB every year worldwide, representing around 12% of all deliveries (Blencowe et al., 2012; Vogel et al., 2018), and resulting in approximately 1.1 million infant deaths annually (Liu et al., 2016). Children born prematurely face an increased risk of complications attributed to multiple organ immaturity, as well as possible lifelong effects on neurocognitive development, visual disorders, and increased risk of chronic disease (Mwaniki et al., 2012). Although PTB is a global public health concern, certain racial and ethnic groups such as African and Asian pregnant women are more predisposed to PTB than others (Beck et al., 2010; Shah et al., 2014; Vogel et al., 2018). Altogether, more than half of PTBs occur in Asia (Beck et al., 2010), but this ethnically-diverse population group is relatively under-studied in terms of PTB causation/risk factors. What is clear is that the combination of environmental influences, high prevalence of infectious disease, and low healthcare resource settings places these women at high risk of adverse pregnancy outcomes following PTB. There is an urgent need for clinically applicable strategies to identify those Asian women in the community at a high risk of PTB. Alongside, knowledge of specific PTB predictive factors for Asian minority groups in other countries will facilitate appropriate medical care in these higher-resource settings.

Pregnancy is an important “formative period” where a series of interconnected physiological and cellular processes aim to support healthy fetal development (Soma-Pillay et al., 2016). These processes include maternal and paternal genetic factors, hormonal changes, immune system modulation, environmental factors, the microbiome and others (Lunde et al., 2007; Workalemahu et al., 2018). While we have known for some time that microbial factors could underpin as many as 50% of all PTBs (Lockwood, 2002; Lamont, 2003), only recently has the association between specific changes in the vaginal microbiome and pregnancy complications started to be unraveled (Nuriel-Ohayon et al., 2016; Fettweis et al., 2019; Serrano et al., 2019). During pregnancy, the composition of the vaginal microbiome undergoes an overall change in microbial diversity and exhibits clade-specific enrichments and depletions (Nuriel-Ohayon et al., 2016; Callahan et al., 2017; Brown et al., 2018; Fettweis et al., 2019; Serrano et al., 2019) that vary according to ethnicity. For example, in Caucasian women, adverse pregnancy outcomes including PTB have been linked with a shift from a *Lactobacilli*-rich vaginal microbiome to a more complex microbial community of *Gardnerella, Prevotella*, and *Lachnospiraceae* family members (bacterial vaginosis (BV)-associated bacterium-I (BVAB-I) (Petrova et al., 2015; Callahan et al., 2017; Fettweis et al., 2019); while African women are less likely to have a vaginal microbiome dominated by *Lactobacillus* species (Ravel et al., 2011; Fettweis et al., 2019).

Similar to the changes observed in the vaginal microbiome composition, maternal immune responses are also modified during pregnancy (Denney et al., 2020). Increase of proinflammatory cytokines such as interleukin- (IL-) 8, IL-1, and IL-6 were shown to trigger early labor in PTB (Donders et al., 2009; Romero et al., 2014). Cytokines may be involved in the pathogenesis of PTB through their role in prostaglandin synthesis and secretion (Romero et al., 1989).

Despite the high burden of PTB in Asian regions, few studies have addressed the composition of the vaginal microbiome and the local immune profiles in reproductive age women living in this geographical area (Yoshimura et al., 2011; Ling et al., 2013; Hong et al., 2016) but not in the context of PTB. Moreover, the high ethnic diversity of the region means that some populations are completely unstudied. This is especially important in areas with low healthcare resources, where an unexpected PTB can have devastating consequences for mother and baby. Therefore, population-specific studies are needed to improve our knowledge of the vaginal microbiome composition during pregnancy and its association with PTB in Asian communities.

Here, we report the results from a study of Karen and Burman ethnicity pregnant women recruited prospectively at 8–14 weeks gestation at the Shoklo Malaria Research Unit (SMRU), Thailand, as part of the larger Molecular Signature in Pregnancy (MSP) study (Brummaier et al., 2019) (**Table 1** and **Supplementary Table 1**). In the study, we compared the vaginal microbiome composition and vaginal cytokine levels of women who experienced PTB, defined as delivery before 37 weeks (n=18), and controls who had a full term birth (TB) (n=36). We measured the vaginal levels of nine cytokines, previous reported to play a role in pregnancy outcomes (Koren et al., 2012; Yockey and Iwasaki, 2018; Fettweis et al., 2019), including: IL-1β, IL-2, IL-4, IL-6, IL-8, IL-10, granulocyte-macrophage colony-stimulating factor (GM-CSF), tumor necrosis factor (TNF)-α, and interferon (IFN)-γ. Combining these longitudinally analyzed data, we went on to generate a predictive vaginal microbial signature that is observed as early as the first trimester of pregnancy and identified a correlative cytokine profile for PTB in these groups of Asian women.

**Table 1:** Description of the cohort included in this paper.

	TB (n=36)	PTB (n=18)	*P*-value*
Age at conception in years; Median (IQR)	24 (21–27)	21.5 (20–24.5)	0.265*
Height at conception in cm mean (IQR)	151.6 (149.2–155.4)	154.15 (150.5–155.6)	0.267*
Weight at conception in kilograms mean (IQR)	48 (44.25–55.25)	48 (42.25–48.875)	0.334*
BMI at conception; Median (IQR)	20.86 (19.33–23.39)	20.125 (18.19 – 20.43)	0.150*
Delivery (%) - Vaginal - Caesarean section	35 (97.2) 1 (2.8)	18 (100) 0 (0.0)	1^‡^
Outcome EGA (days); Median (IQR)	276.5 (269.75–283)	253.5 (242–254.75)	<0.001*
Birth weight in grams (IQR)	3060 (2,907.5–3,310)	2265 (1,980–2440)	<0.001*

*Mann Whitney U Test; ^‡^Fisher Exact test.

EGA, Evaluation of Gestational Age; IQR, InterQuartile Range.

PTB, preterm birth; TB, term birth.

## Materials and Methods

### Study Design and Participants

This observational, prospective, pregnancy-delivery-postpartum cohort enrolled pregnant women with no prior adverse obstetric or overt medical history and was a collaboration between Sidra Medicine, Doha, Qatar, and SMRU, Mae Sot, Thailand (Brummaier et al., 2019). SMRU is a field station of the Faculty of Tropical Medicine, Mahidol University, Bangkok, Thailand, and is part of the Mahidol-Oxford Research Unit, which combines research and humanitarian work to serve rural and disadvantaged migrant and refugee populations on the Thailand-Myanmar border. The study was conducted in accordance with the Declaration of Helsinki and followed ICH Guidelines for Good Clinical Practice.

### Participant Recruitment, Clinical History, and Sample Collection

**Figure 1:**
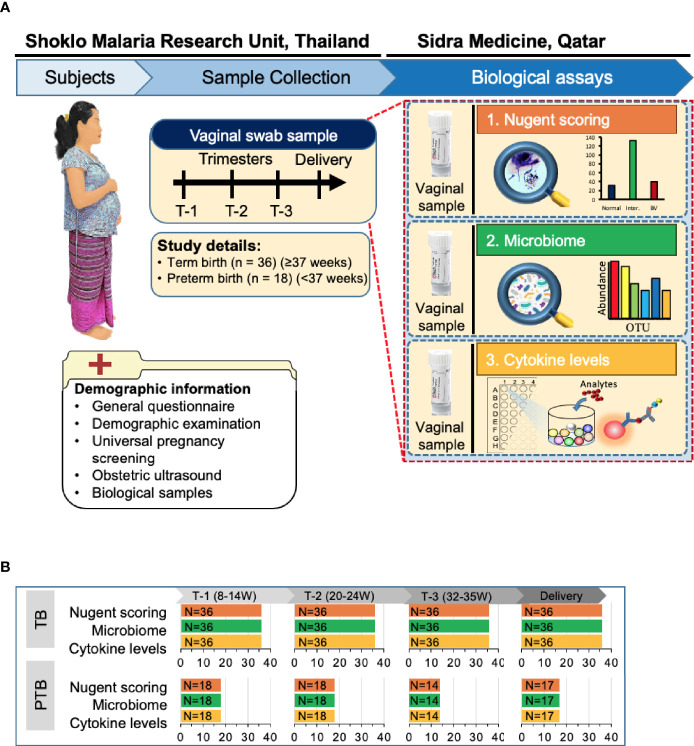
Overview of the study design. **(A)** Of the 400 Karen and Burman women enrolled in Molecular Signature in Pregnancy (MSP) study, 18 pregnant women experienced PTB (pre-term birth) and 36 women who delivered at term (TB) were selected for this study. Vaginal swabs were taken from these women at Shoklo Malaria Research Unit (SMRU) health care clinics at each trimester of pregnancy: T-1 (8–14 weeks), T-2 (20–24 weeks), T-3 (32–35 weeks) and at delivery. Detailed demographic information was collected at the first visit. Vaginal swab samples were used for Nugent scoring to determine bacterial vaginosis status, for microbiome analysis and for assessing the levels of different cytokines. BV, bacterial vaginosis; OTU, operational taxonomic unit; W, weeks of gestation. **(B)** Number of samples collected from women in both groups and processed for each analysis.

First trimester pregnant women with a viable, singleton pregnancy were enrolled at SMRU’s antenatal care (ANC) clinics on the Thailand-Myanmar border. Gestational age was determined by early ultrasound scan and women with age between 18–49 years with an estimated gestational age from 8 weeks 0 days to 13 weeks 6 days (T1) at the time of enrollment were included in the study. Enrollment of pregnant women and collection of samples, including the inclusion and exclusion criteria were described earlier (Brummaier et al., 2019). At the time of recruitment, comprehensive maternal demographic information, medical, and obstetric history were recorded together with a detailed physical and obstetric examination. Women were followed-up each trimester (T2: 20-24 weeks, T3: 32-35 weeks) and during delivery (**Figure 1**).

Vaginal swab samples were collected from the posterior formix at a sampling point in each trimester, and at delivery, by a trained midwife using the Copan Eswab™ collection system. Three swabs were taken at each timepoint: one to extract genomic bacterial DNA, one to prepare a Gram stain smear to assess the Nugent score, and one for measurement of vaginal cytokines. Samples were transferred daily from the clinic sites to the central laboratory facility and stored at -80 degrees Celsius. For international shipment samples were kept on dry ice in Styrofoam boxes that were equipped with temperature monitors.

The MSP cohort consisted of 381 pregnant women, including 19 who experienced PTB (Brummaier et al., 2020). One PTB participant was excluded from the study, due to the non-availability of the sample set. The case matching of the remaining 18 PTB participants was performed with control participants who delivered at term (≥37 weeks) based on age, parity, and gravida (Koullali et al., 2020).

### Nugent Scoring

Vaginal swab smears were screened for BV using the Nugent scoring method described earlier (Eschenbach et al., 1988). Briefly, frozen vaginal swab samples were thawed on ice, vortexed vigorously for 1 min then swabs were rolled onto slides, air dried and Gram-staining using the standard protocols. Vaginal smears were evaluated using the Nugent scoring method by the same trained individual for all time points to assess the relative abundance of three types of bacterial cell morphotypes: large Gram-positive rods (*Lactobacillus* morphotypes), small Gram-negative rods, and cocci (*Gardnerella vaginalis*, Bacteroides), and curved Gram-negative bacilli (Eschenbach et al., 1988). The slides were evaluated in triplicates by three individuals and average score was reported. The Nugent scores range from 0–3 (normal), 4–6 (intermediate), and 7– 10 (indicative of BV) (Nugent et al., 1991).

### DNA Extraction and 16S rRNA Gene Sequencing

The total DNA from vaginal swabs was extracted using the modified protocol MoBio Powersoil modified Method #3 as previously published (Mattei et al., 2019). DNA concentration was measured using Nanodrop.

The V1-V3 regions of the 16S rDNA were amplified using forward primers: 27F with 12 bp golay barcodes containing a specific Illumina 5’ adapter for each sample and a common reverse primer 515 R (Mattei et al., 2019). In brief, PCR was performed in triplicate in a 50 μl reaction mixture containing 10 ng of template DNA and 2x Phusion HotStart Ready Mix. The following thermal cycling conditions were used: 5 min of initial denaturation at 94°C; 25 cycles of denaturation at 94°C for 30 s, annealing at 62°C for 30 s, and elongation at 72°C for 30 s; and the last step at 72°C for 10 min. The amplified PCR products of approximately 650 bp in size from each sample were pooled in equimolar concentrations. This pooled PCR product was purified using AgenCourt AMPure XP magnetic beads. High throughput sequencing was performed on an Illumina MiSeq 2 × 300 platform (Illumina, Inc. San Diego) in accordance with the manufacturer’s instructions. Image analysis and base calling were carried out directly on the MiSeq.

### Vaginal 16S rRNA Taxonomic Profiling

Sequenced data were demultiplexed using MiSeq Control Software (MCS) then quality controlled using FastQC (Andrews, 2010). Forward and reverse end sequences of respective samples were merged through the PEAR tool (Zhang et al., 2014) and sequence reads of quality score < 20 were discarded. All merged reads were trimmed to 160bp>Reads<500bp using the Trimmomatic tool (Bolger et al., 2014). Trimmed FASTQ files were converted into FASTA files. Demultiplexed FASTA files were analyzed using QIIME (Quantitative Insights Into Microbial Ecology) v1.9.0 pipeline (Caporaso et al., 2010). Operational taxonomic units (OTUs) were generated by aligning against the SILVA database.

### Microbial Diversity Analysis

Alpha diversity was measured by R software, using the phyloseq package (McMurdie and Holmes, 2013). Beta diversity was represented using Phylogenetic beta diversity metrics (Lozupone et al., 2007) and the differences in the beta diversity were presented as principal coordinate analysis using the QIIME v1.9.0 pipeline. We then performed a longitudinal analysis to evaluate the relationship between vaginal microbial community, delivery status, and the stage of pregnancy using the ggplot package in the R software.

### Vaginal Cytokine Profiling

Cytokine levels in the vaginal swab samples were quantified using Bio-Rad Bio-Plex Pro human cytokine 8-Plex assay kit (Bio-Rad Laboratories, Inc., USA) with a Luminex 3D system. In addition, a single-Plex assay was used for IL-1B (Bio-Rad Laboratories, Inc., Hercules, CA, USA) (Sharma et al., 2009). Frozen vaginal swab samples were thawed, vortexed vigorously for 1 min and the swab squeezed on the wall of the tube to maximize elution. The swab samples were then centrifuged at 700 x g for 10 min. The Bio-Rad assay was performed using samples and serial dilutions of standards in duplicate, according to the manufacturer’s instructions. Cytokine levels were analyzed using LuminoXponent software. Chemokine and cytokine values for samples with levels below the lower limit of detection (LOD) were assigned the lower limit of detection for the specific cytokine. The lower limits of detection ranged from 0.01 to 1.1 pg/ml. The median proportion of samples below the LOD was 1.85% (ranging from 0% to 25.4%).

### Statistical Analysis

Statistical significance of alpha diversity measures such as Observed, Chao1, Shannon and Simpson indices were calculated using minitab17 (Minitab statistical software). *P*-values lower than 0.05 were considered statistically significant. Adonis was used to calculate the distance matrix difference between the categories included in this study using unweighted beta diversity parameters (Lozupone et al., 2007).

For the cytokine data analysis, all cytokine values were log-transformed before analysis to achieve normality and homogeneity of the raw values. The unpaired *t*-test analysis with Welch’s correction was used to determine significant differences between cytokine levels of women who experienced PTB versus TB. Statistical analyses were performed using GraphPad Prism 8 (GraphPad Softwares Inc. USA).

Association between cytokines profile and vaginal microbiome data was performed using sparse canonical correlation analysis (sCCA) was performed as described earlier (Witten et al., 2009; Fettweis et al., 2019). Briefly, an integrative analysis of log-transformed cytokine data and 16S rRNA vaginal taxonomic data was performed to explore the correlation between two data sets of quantitative variables measured on the same subjects using sCCA. The most abundant bacterial taxa were designated as present if they comprised ≥0.1% of the total vagitype profile in either group, and nine cytokines were selected for the analysis. For TB and PTB, sCCA was performed separately using the sgcca package in R and displayed in a correlation circle plot (Rohart et al., 2017). All variables with a strong positive correlation are grouped together, while variables with negative correlations are plotted opposite each other.

Predictive modeling of PTB using early pregnancy microbiome and cytokines profiles was performed using the first trimester profiles of *L. crispatus, L. iners, P. buccalis*, *Finegoldia*, and all cytokines. The microbiome profile and cytokines levels were normalized using log unit method and cross-validated before the machine learning step. The construction of machine learning model was performed using Elastic Net (ENet) classifier, which is the combination of the lasso penalty and the alternative ridge (or L2) penalty, and it was trained and enforced at least five non-zero coefficients to measure the performance of the model through sensitivity and specificity threshold scores (Zeller et al., 2014). The area under the ROC curve (AUC) results were considered excellent for AUC values between 0.9–1, good for AUC values between 0.8–0.9, fair for AUC values between 0.7–0.8, poor for AUC values between 0.6–0.7 and failed for AUC values between 0.5–0.6 (El Khouli et al., 2009).

## Results

### Description of the Cohort

Clinical, demographic, and pregnancy outcome characteristics data of women included in this study are summarized in **Table 1**. Vaginal swab samples were analyzed from 54 women (18 women who experienced PTB and 36 age-matched women who experienced TB) at first, second, third and delivery (**Figure 1**). At the time of enrollment into the cohort, there were no significant differences in maternal height, weight, body mass index or delivery mode between PTB and TB groups (**Table 1** and **Supplementary Table 1**). The mean gestational age at delivery for those who delivered PTB and TB was 36.2 and 39.5 weeks respectively, and as expected, preterm neonates had a lower birth weight compared to term neonates (**Table 1**).

### PTB Is Positively Associated With Bacterial Vaginosis in Burman and Karen Women

**Figure 2:**
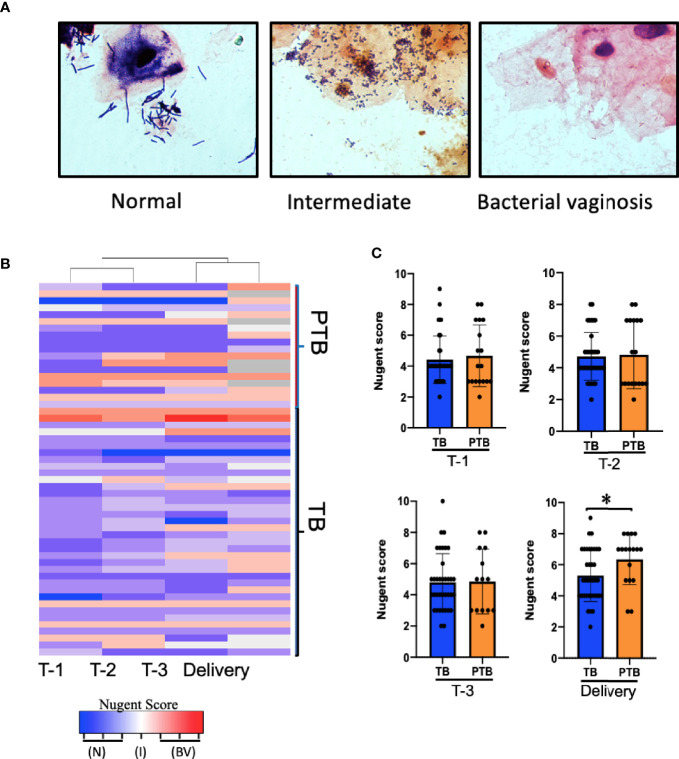
Association between Nugent score and PTB. **(A)** Representative images of Gram-stained vaginal swabs with scoring normal, intermediate, and BV, Slides were evaluated under 1,000x magnification according to the 10-point Nugent scale. **(B)** Heatmap showing the Nugent scores of individual women who delivered at term (TB) and women who experienced pre-term birth (PTB). Columns represent the trimester of pregnancy. Each row represents one subject. **(C)** Comparison of the average Nugent score in the TB or PTB groups during pregnancy. *P*-value was calculated using unpaired *t*-test (two-tailed) with Welch’s correction for difference in Nugent score between TB and PTB groups. *p < 0.05. T-1: Trimester-1, T-2: Trimester-2; T-3: Trimester-3; N, Normal; I, Intermediate; BV, Bacterial Vaginosis.

In Caucasian women, a healthy vaginal microbiome is generally dominated by *Lactobacillus species* such as *L. crispatus* (Ravel et al., 2011; Serrano et al., 2019); while an imbalanced microbiome (termed bacterial vaginosis (BV)) is characterized by a decrease in *Lactobacillus species* and an increase in mixed anaerobes such as *Gardnerella*, *Atopobium*, *Prevotella, Megasphaera* species, and others (Donders et al., 2000; Brocklehurst et al., 2013). As it is unknown whether the same species characterize vaginal microbial balance/imbalance in this group of Asian women, we began by identifying and comparing the bacteria present in vaginal swab smears from our TB and PTB cohorts. We used Nugent scoring, which is based on microscopic morphotype enumeration of Gram-positive *Lactobacilli* vs. Gram-negative bacteria, to define normal (score = 0–3), altered/intermediate (score = 4–6), or BV (score = 7–10) categories (Nugent et al., 1991). Representative images for each category are shown in **Figure 2A**. Women in the PTB group were more likely to exhibit either high or low Nugent scores during pregnancy than women in the TB group, of which the majority exhibited Nugent scores in the intermediate category (**Figures 2B, C** and **Supplementary Figure 2**). However, by delivery 12 of the 18 PTB women exhibited BV, leading to a significantly higher average Nugent score compared to those in the TB group (**Figure 2C**, fourth panel). This is consistent with the previously-reported positive association between PTB and BV in a predominantly Caucasian cohort of women (DiGiulio et al., 2015), but is the first evidence of a similar association in an Asian cohort.

### High Microbial Diversity Characterizes the Vaginal Microbiome in PTB

**Figure 3:**
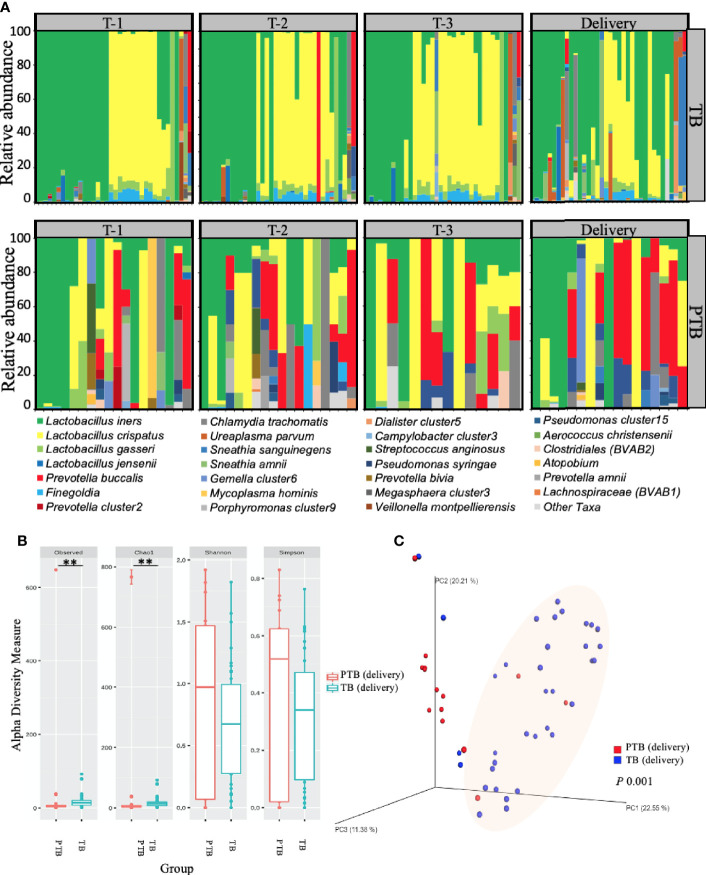
Vaginal microbiome composition in women with TB and PTB. **(A)** Stacked bar plots showing the relative abundance (%) of each microbial species in vaginal swabs from women who had full term birth (TB) and women who experienced preterm birth (PTB). Each vertical bar represents one woman. **(B)** Alpha diversity of the microbiome at delivery was compared between the two groups by the number of operational taxonomic units (OTUs) observed and by the Chao1, Shannon and Simpson diversity indices. The asterisks indicate a significant difference in diversity of microbial communities between the two groups (**P < 0.01). **(C)** Beta diversity plot showing microbial communities clustered using Principle Coordinates Analysis (PCoA) based on Bray–Curtis dissimilarities between vaginal microbiomes. Statistical significance of the alpha diversity measures was calculated using the Kruskal-Wallis test for non-parametric data, while analysis of similarities method (ANOSIM) was used for calculation of the distance matrix difference between the TB and PTB groups using unweighted beta diversity parameters. P-values lower than 0.05 were considered statistically significant.

Having established that increased bacterial diversity/BV was positively associated with PTB in our cohort, we next asked which bacterial species were involved. To assess the detailed changes in microbiome composition we generated vaginal microbiota relative abundance profiles using 16S rRNA taxonomic analysis. Despite being genetically different (Summerer et al., 2014), both Karen and Burman ethnic women showed similar vaginal microbiome (**Supplementary Figure 1**), however when we compared the vaginal microbial profile women experienced PTB with women experienced TB, we observed a clear differences at both the phylum and species levels (**Figure 3** and **Supplementary Figure 3**).

Given the apparent variability in distribution of different microbial species between TB and PTB groups, we next applied diversity analysis to understand whether the variability itself was important. We applied two diversity analyses: alpha diversity (**Figure 3B**) measures the average species diversity within a sample community and was calculated first from the total number of unique operational taxonomic units (OTUs) observed per sample; then using the Chao1 abundance-based richness estimator, which is sensitive to rare OTUs (Chao, 1987); and lastly by Shannon (Shannon, 1948) and inverse Simpson (InvSimpson) (Simpson, 1949) approaches which are more dependent on highly-abundant OTUs and less sensitive to rare OTUs (McMurdie and Holmes, 2013). Following alpha analysis, we then employed beta diversity to understand the divergence in community composition between samples (**Figure 3C**), which we assessed using principal coordinate analysis (PCoA) based on Bray-Curtis dissimilarities (Lozupone et al., 2007). When we compared the overall vaginal microbial richness at delivery by alpha diversity analysis, we saw that women who delivered preterm showed significantly more microbial richness (Observed, p*=*0.005; Chao1, p=0.005); coupled with greater beta diversity (p=0.001) compared to women who had term deliveries (**Figures 3B, C**).

**Figure 4:**
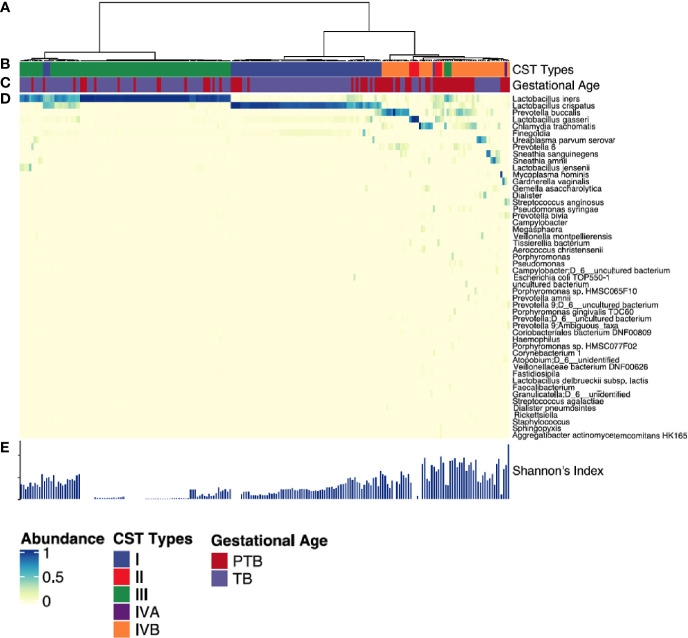
Vaginal microbiota profiles of women who had PTB and TB deliveries. **(A)** Hierarchical clustering of Euclidean distance matrices with Ward linkage on relative abundances of reads for each OTU within individual vaginal swab samples collected at all time points. **(B)** Community state types (CST) identified across all the study subjects. Each CST is represented by a different color according to the key shown underneath. **(C)** Gestational age category (PTB shown in red, TB shown in blue). **(D)** Heatmap of relative abundances of bacterial species within the vaginal microbiota of each woman. Each column represents a woman’s vaginal microbiota profile, and each row represents a bacterial species. Only species that represent at least 0.5% of the total microbiome in at least one sample are shown. **(E)** Shannon diversity indices calculated for each sample. Each CST is represented by a different color: CST-I, *Lactobacillus crispatus*-dominated; CST-II, *Lactobacillus gasseri*-dominated; CST-III, *Lactobacillus iners*-dominated; CST-IVA, lower abundance of *Lactobacillus spp* together with low proportions of anaerobic bacteria such as *Anaerococcus, Corynebacterium, and Streptococcus*; CST-IVB: dominated by higher abundance of the genera *Atopobium, Prevotella, Parvimonas, Sneathia, Gardnerella, Mobiluncus*, or *Peptoniphilus* and several other taxa.

**Figure 5:**
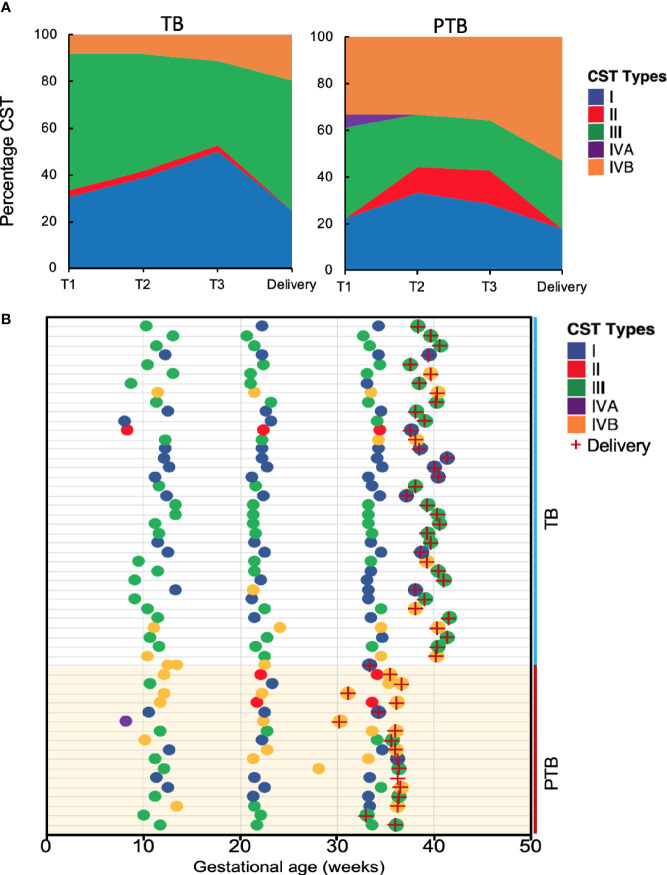
Vaginal community state types during the course of pregnancy. **(A)** Stacked area charts of community state type (CST) showing the dynamics of the vaginal microbiome in the full term birth (TB) and preterm birth (PTB) groups at the three trimesters of pregnancy (T-1, T-2, T-3) and at delivery. X-axis represents the gestational age T-1: Trimester-1; T-2: Trimester-2; T-3: Trimester-3, y-axis represents the percentage of each CST in the samples from each group. **(B)** Profiles of community state type (CST) for pregnant women who delivered at term (TB) and those who had preterm birth (PTB) as a function of gestational age. Delivery is indicated by a red cross.

We then clustered vaginal microbial diversity using Euclidean distance matrices with Ward linkage (**Figure 4**) into the following community state types (CST), as previously described by Ravel et al: CST-I (*Lactobacillus crispatus*-dominated), CST-II (*Lactobacillus gasseri*-dominated), CST-III (*Lactobacillus iners*-dominated), CST-IVA (lower abundance of *Lactobacillus spp*, together with low proportions of various anaerobic bacteria such as *Anaerococcus*, *Corynebacterium*, *Finegoldia*, or *Streptococcus*); CST-IVB (higher abundance of the genera *Atopobium*, *Prevotella*, *Parvimonas*, *Sneathia*, *Gardnerella*, *Mobiluncus*, or *Peptoniphilus* and several other taxa often associated with high Nugent scores); and CST-V (*Lactobacillus jensenii*-dominated) (Ravel et al., 2011; Ma and Li, 2017). The most commonly observed was CTS III (*L. iners*) followed by CST I (*L. crispatus*) and CST IVB (dominated by *Prevotella Buccalis*) respectively (**Figure 4**). Comparing TB and PTB groups confirmed substantial differences in the overall microbial profiles, with TB women typically exhibiting a higher prevalence of *Lactobacillus*-dominated vagitypes (CST-I and -III), and PTB women exhibiting a significantly higher frequency of CST-IV (p<0.0001) (**Figure 5A**). We then looked at the dynamics of the vaginal microbiome during pregnancy to assess whether the vaginal microbial communities in TB or PTB groups persist across the sampling points or whether there is a transition between different vagitypes during pregnancy. We observed that most women in the TB group had a vaginal environment dominated by either CST-III (50%) or CST-I (36%) throughout the pregnancy period (**Figure 5A**), while, women in the PTB group had a higher prevalence of CST-IV vagitype (40%) as early as the first trimester (**Figure 5A**). The profiles of community state types (CSTs) for each pregnant woman as a function of gestational time clearly highlight the enrichment of CST-IVB in women in the PTB group (**Figure 5B**). Similar associations between higher levels of CST-IV and lower levels of *Lactobacillus species* and PTB were also observed in a predominantly African cohort of women (Fettweis et al., 2019), highlighting that their abundance might have some conserved influence on pregnancy outcomes.

Taken together, these data show that the vaginal microbiome associated with TB in this Asian cohort is generally dominated by CST-III throughout the course of pregnancy. In contrast, PTB is significantly associated with CST-IVB, characterized by a lower abundance of *Lactobacillus species* and a higher abundance of mixed anaerobic bacteria known to be associated with high Nugent scores (Eschenbach et al., 1988; Donders et al., 2000), as well as with greater overall microbial diversity across the PTB group. These effects were present throughout pregnancy starting from the first trimester.

### Low *L. crispatus*, Low *Finegoldia*, and High *P. buccalis* Precede PTB in Burman and Karen Women

**Figure 6:**
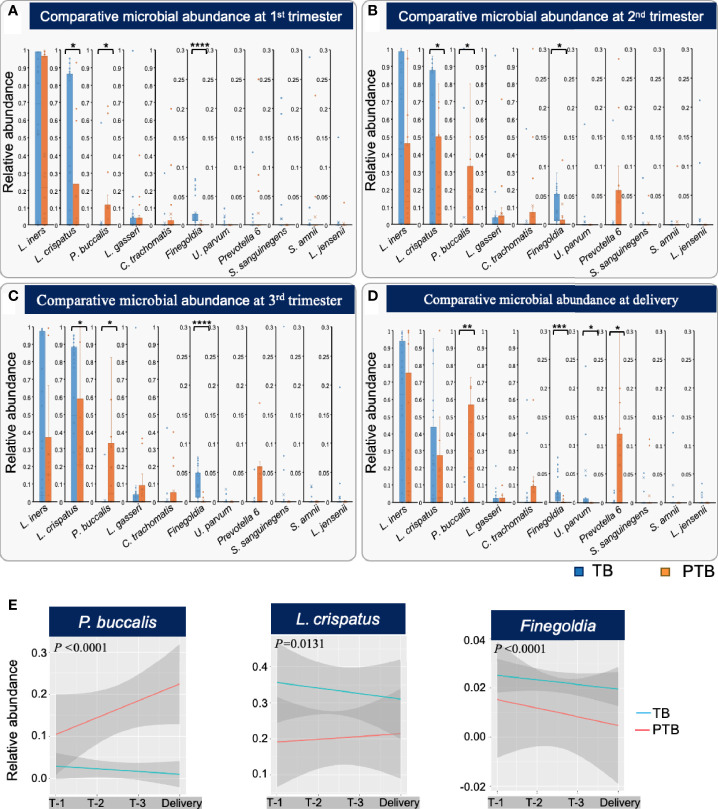
Different bacterial taxa associated with TB and PTB. **(A)** Differences in relative abundance of the top 11 microbial taxa found between full term birth (TB) and preterm birth (PTB) groups in the first trimester; **(B)** second trimester; **(C)** third trimester, and **(D)** at the time of delivery. Blue bars represent the TB group, while orange bars represent PTB. The asterisks indicate a significant difference between two groups (*P < 0.05, **P < 0.01, ***P < 0.001, ****P < 0.0001). **(E)** Longitudinal trends in relative abundance of the statistically significant microbial taxa identified by comparing TB and PTB groups, analyzed using ggplots. Blue lines represent the TB group and red lines the PTB group. The *P-*values were calculated using the unpaired *t*-test (two-tailed) with Welch’s correction for difference in proportional microbial abundance between TB and PTB groups.

Given the high diversity of microbes in samples from women who experienced PTB, we next asked whether any microbial species were particularly associated with premature delivery. We first compared the relative levels of the eleven most abundant bacterial taxa (**Figure 4**) between the TB and PTB groups (**Figure 6**). Of these, three were significantly different: in PTB women, *P. buccalis* was significantly more abundant throughout pregnancy from as early as the first trimester, compared to women with TB (**Figures 6A–D**); while *Finegoldia* and *L. crispatus* were significantly less abundant during the pregnancies of PTB women compared to TB women, also as early as the first trimester (**Figures 6A–D**). These data are similar to earlier findings in women from African ancestry showing that lower levels of *L. crispatus* in early and mid-pregnancy are associated with PTB (Fettweis et al., 2019).

We further confirmed the importance of longitudinal trends in abundance of the three most significant bacteria using ggplots incorporating delivery status (TB or PTB), longitudinal microbial abundance and timepoint during pregnancy (**Figure 6E**). We observed that women in the PTB group exhibited a significant increase in relative abundance of *P. buccalis* (*P*<0.0001), whereas women who delivered at term showed a significant increase in *L. crispatus* (*P*=0.0131), and *Finegoldia* (*P<0.0001*) from trimester 1 and throughout pregnancy (**Figure 5E**). In summary, we have identified early trends in abundance of specific bacterial taxa that occur during pregnancies that end in PTB, and distinct trends that characterize TB pregnancies: these trends are evident as early as the first trimester, indicating their possible predictive potential.

### Vaginal Cytokines Differ Between PTB and TB Pregnancies

**Figure 7:**
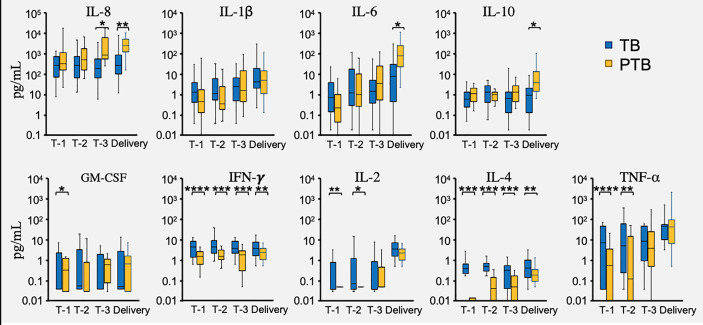
Vaginal cytokine levels during PTB and TB pregnancies. Cytokines were measured in fluid used to elute vaginal swabs from full term birth (TB) and preterm birth (PTB) women in the three trimesters of pregnancy (T-1, T-2, T-3) and at delivery. Blue bars represent the cytokine levels measured in the TB group, while the orange bars represent the cytokine levels in the PTB group. *P*-values were calculated using the unpaired *t-*test with Welch’s correction. The Y axis represent the cytokine levels in log scale. The asterisks indicate a significant difference in cytokine levels between the two groups (*P < 0.05, **P < 0.01, ***P < 0.001 and ****P < 0.0001).

The vaginal microbial environment is maintained by a delicate interplay between host factors (ethnicity, genetics and immune mediators) and microbial biology. While altered vaginal cytokine patterns have been described during pregnancy (Yockey and Iwasaki, 2018), their relationship to the vaginal microbiota and pregnancy outcomes is incompletely defined. In this study, we measured the vaginal levels of nine cytokines, including: IL-1β, IL-2, IL-4, IL-6, IL-8, IL-10, GM-CSF, TNF-α, and IFN-γ (Yockey and Iwasaki, 2018; Fettweis et al., 2019), and asked whether these levels differed between TB and PTB groups. In PTB pregnancies we found significantly higher levels of IL-8 in trimester 3 samples compared to TB pregnancies at the same timepoint; while by the point of delivery IL-8, IL-6 and IL-10 were all higher in the PTB group (**Figure 7**). In contrast, women with PTB showed significantly lower levels of TNF-α and IL-2 in the first two trimesters than did TB women (**Figure 7**). However, the most robust differences were seen in levels of IFN-γ and IL-4 which were significantly lower in the PTB group throughout their entire pregnancies and at delivery (**Figure 7**). Thus, we have identified several potential biomarkers in the vaginas of PTB women that distinguish their pregnancies from those of TB women, from as early as the first trimester.

### Integrative Analysis of Vaginal Cytokine Levels and Microbial Profiles

**Figure 8:**
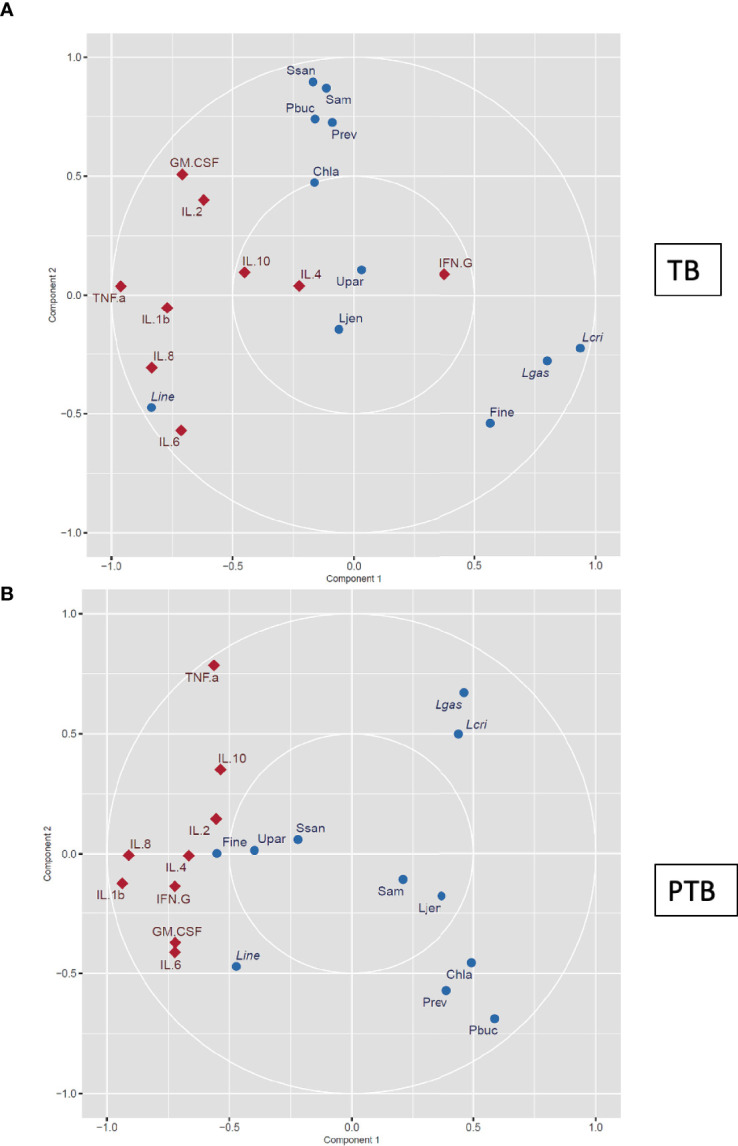
Canonical correlation analysis of vaginal microbial signature and cytokine levels. The vaginal microbial taxonomic profiles and cytokine levels in samples collected from women who experienced **(A)** TB and **(B)** PTB at the first trimester (8–14 weeks) were log-transformed and co-integrated using canonical correlation analysis. Cytokines are represented as red diamonds, and bacteria are represented as blue circles. Positively correlated variables are grouped together, while negatively correlated variables are positioned on opposite sides of the plot origin. Thus cytokines or microbial taxa that are clustered tightly are highly correlated, and factors that are distant from each other are not correlated. Pbuc, *Prevotella buccalis*; Fine, *Finegoldia*; Chla, *Chlamydia trachomatis*; Lcri, *Lactobacillus crispatus*; Line, *Lactobacillus iners*; Lgas, *Lactobacillus gasseri;* Ljen, *Lactobacillus jensenii;* Prev, *Prevotella* 6; *Ssan*, *Sneathia sanguinegens*; Upar, *Ureaplasma parvum; Sam*, *Sneathia amnii*.

To bring together our data on the vaginal microbiome and cytokine environment linked with PTB in our cohort of Burman and Karen Asian women we performed an integrative sCCA which assessed the association between the abundance of the main microbial taxa, the levels of cytokines, and pregnancy outcomes. For each participant, the samples collected at the first trimester (8-14 weeks) were characterized. In the women who delivered at term, we observed a moderate positive correlation between *L. crispatus*, *L. gasseri*, and *Finegoldia* as well as a strong negative correlation between *L. crispatus*, *L. gasseri*, *Finegoldia*, and several taxa known to be associated with dysbiosis including *C. trachomatis*, *P. buccalis, Prevotella, S amnii*, *S sanguinegens* (**Figure 8A**). We also observed a strong negative correlation between *L. crispatus*, *L. gasseri*, *Finegoldia*, and *L. iners* (**Figure 8A**).

In contrast, in women who experienced PTB, the abundance of *L. crispatus* and *L. gasseri* was negatively correlated *with Finegoldia*, and similar negative correlation was observed between *L. crispatus*, *L. gasseri* with *C. trachomatis*, *P. buccalis* and *Prevotella* (**Figure 8B**). Similar to the TB group, a strong negative correlation between *L. crispatus*, *L. gasseri*, and *L. iners* was observed (**Figure 8B**). While *P. buccalis* and other taxa known to be associated with dysbiosis were negatively correlated with the cytokines tested, abundance of *Finegoldia* was strongly correlated with levels of IL-2 and IL-4 in the PTB group, and this was observed as early as the first trimester (**Figure 7B**). To further evaluate the early predictability of proposed microbial signature and cytokine levels, we used the ENet model. The resulting model incorporates the four taxa: *L. crispatus, P. buccalis, L. iners*, and *Finegoldia*, which all were differentially present in PTB and TB groups in addition to the cytokines levels measured in the first trimester. The predictive model has an expected sensitivity of 88.3%, specificity 86.6% and an area under the receiver operating characteristic (ROC) curve of 0.861.

On the other hand, when we performed the sCCA integrative analysis at the time of delivery, a strong positive correlation was observed between pro-inflammatory cytokines such as IL-1b, IL-6, and IL- 8 with *P. buccalis* and *Prevotella 6*, but a strong negative correlation of the same cytokines with *L. crispatus* was observed (**Supplementary Figure 4**).

In this study, we identified a predictive vaginal microbiota signature for PTB that was detectable as early as the first trimester of pregnancy, characterized by higher levels of *P. buccalis*, and lower levels of *L. crispatus and Finegoldia*, accompanied by decreased levels of cytokines including IFNγ, IL-4, and TNFα.

## Discussion

The women enrolled in this study had no prior adverse obstetric or overt medical history, and therefore represented the women in whom a predictive signature would be of highest value in identifying those apparently low-risk individuals at increased risk of unanticipated PTB. Our data revealed that this cohort of Asian women may have a different vaginal microbial composition compared to women of European and American ancestries (Walther-Antonio et al., 2014; MacIntyre et al., 2015; Chawanpaiboon et al., 2019). Although the cause of these differences remains unclear, both genetic and environmental factors, including geographic location, diet, age, BMI (Body mass index), drug exposure, physical activities, and availability of resources such as access to medical care are likely to contribute (Conlon and Bird, 2014; Kumar et al., 2018; Singh et al., 2019; Kumar et al., 2020; Verwijs et al., 2020). Thus, our results confirm the need for ethnicity-specific studies to identify microbial signatures associated with healthy and complicated pregnancies, including the risk of PTB.

While a normal Nugent score is always thought to be accompanied by a healthy pregnancy outcome, our data show that 60% of Asian women in the TB group had an intermediate Nugent score throughout their pregnancy: thus, in these women, an intermediate Nugent score was not associated with adverse outcome. The high prevalence of intermediate scoring might be due to the higher counts of *L. iners* (Jespers et al., 2012) in their vaginal microbiome, which has been previously reported as the most dominant *Lactobacillus* spp. detected in Asian women (Ravel et al., 2011). The presence *L. iners* in the vaginal microbiome has been also strongly associated with an intermediate transient microbiota characterized by an intermediate Nugent score of 4–6 (Shipitsyna et al., 2013). A previous study on Caucasian women reported that women dominated by *L. iners* during the first trimester of pregnancy were 10 times more likely than those carrying other *Lactobacilli species* to transition to a dysbiotic microbiome during pregnancy and to deliver preterm (Petricevic et al., 2014), but we do not see this here. So while there is still a lot of controversy over whether *L. iners* is more likely a friend or a foe overall, our study reports for the first time that *L. iners* is associated with healthy pregnancy outcomes in an Asian cohort.

Although there are several studies that have assessed PTB in other ethnicities (Ravel et al., 2011; Petricevic et al., 2014; Fettweis et al., 2019; Serrano et al., 2019), Asian populations in general have been relatively under-studied, and this is the first study to be conducted on the composition of the vaginal microbiome in Karen and Burman pregnant women and its relation to delivery at full term or preterm. Our data show that in TB women there was a transition in the vaginal microbiome between two major CSTs: CST-1 (*L. crispatus*) and CST-III (*L. iners*) with a minimal representation of other CSTs throughout pregnancy. In contrast, in PTB women, we found a decrease in the abundance of *L. crispatus* and *Finegoldia* coinciding with an increase in the prevalence of CST-IVB taxa, particularly *P. buccalis*. This differential microbial signature was detectable as early as the first trimester and was positively correlated with the high Nugent score observed in the PTB group. *Finegoldia* is usually considered a member of CST-IVA when present in combination with a modest proportion of *Lactobacillus species*, and a low proportion of *Anaerococcus*, *Corynebacterium* or *Streptococcus* (Gajer et al., 2012; Ma and Li, 2017): perhaps due to the relatively lower abundance of the remaining members, CST-IVA was minimally detected in our cohort and *Finegoldia* was mostly represented by itself. It is also worth mentioning that CST-IVA is often associated with a low Nugent score (Ma and Li, 2017).

All women enrolled on the study were considered low risk for pregnancy complications including pre-term birth. Accordingly, the majority of deliveries in both PTB and TB cohorts occurred vaginally after spontaneous onset of labor or rupture of membranes, which indicates that the local vaginal environment or production of proinflammatory cytokines in the lead up to labor could play a role in modulation of local vaginal environment and thereby risk of PTB. Given that one of the main aims of this study was to generate a predictive PTB signature, we measured the levels of local cytokines in both groups throughout pregnancy. Our data showed that levels of both IL-4 and IFN-γ were lower in the PTB group compared to women who delivered full term, and this was evident from the first trimester of pregnancy. We also observed that vaginal IL-6 and IL-8 levels were significantly increased during trimester-3 or before delivery in the PTB group. Increased expression of proinflammatory cytokines, including IL-8, IL-6, and TNF-α has been previously reported (Fettweis et al., 2019) and is predicted to play a role in the induction of labor.

Our correlation analysis at the earliest gestational time also revealed two microbial scenarios according to pregnancy outcome: in women who delivered full term, *L. crispatus*, *L. gasseri*, and *Finegoldia* were negatively correlated with *L. iners* and with the dysbiotic taxa from the CST-IVB; *L. iners* abundance was also negatively correlated with the dysbiotic taxa. Women who delivered preterm also exhibited a negative correlation between *L. crispatus* and *L. gasseri* with the dysbiotic taxa and with *L. iners*; but here they were also negatively correlated with *Finegoldia*. This was further confirmed by the fact that the relative abundance of *Finegoldia* in the PTB group was significantly lower compared to the TB group throughout pregnancy. *Finegoldia* was positively loosely correlated with IL-2 and IL-4, both higher in women who delivered full term. Thus, our findings are consistent with earlier observations, where vaginal dysbiosis, including elevated levels of CST-IV taxa (for example *P. buccalis* and *C. trachomatis*), have been associated with bacterial vaginosis to drive the adverse pregnancy outcomes, including PTB (Fredricks et al., 2005; O’Connell and Ferone, 2016; Brown et al., 2018; Fettweis et al., 2019). *P. buccalis* is a Gram negative bacterium that may stimulate the release of pro-inflammatory cytokines *via* the interaction with the Toll-like receptors (TLR) present on the immune cells and can in turn promote the release of prostaglandins and proteases (Larsen, 2017). In contrast, *L. crispatus*-rich vaginal microbiome has been associated with long-term reproductive health, growth of the fetus and normal pregnancy (MacIntyre et al., 2015; Amabebe and Anumba, 2018).

Our findings have several strengths: first, they allow the prospective identification of apparently low-risk women who actually carry a high risk of PTB - in low-resource settings early identification of PTB risk has a real chance of improving the outcome for mother and child by ensuring that they receive extra monitoring and deliver in a medical setting. Secondly, these data are more broadly applicable to Asian women in ethnically-diverse populations in high income nations, such as Qatar and Singapore, where Burman and Karen women might well be a minority group and specific knowledge of their microbiome could be useful; and thirdly, this study represents an important step toward understanding microbiome-induced complications and exploring the potential of developing personalized anti-microbial therapies targeting specific bacteria and aiming to reduce the risks of PTB. Previous studies have included a minority of Asian women, but this unique signature has so far been “lost in the noise”. However, the question remains whether the vaginal microbiota is the direct cause of high PTB risk, or if there is something else that drives the dysbiosis as well as the PTB? Could it be that immune dysregulation, leading to the observed difference in vaginal cytokine levels is actually the reason both vaginal microbial dysbiosis and subsequently high PTB risk? These could be interesting avenues for investigation for future research.

## Conclusions

In this paper, we show that higher levels of *P. buccalis* accompanied by lower levels of *L. crispatus* and *Finegoldia* in Asian pregnant women represent a predictive PTB signature that could be used as early as the first trimester to identify those most at risk to develop PTB. Further studies are required to determine the mechanism by which this microbial signature increase the risk of PTB and contribute to its pathophysiology. More broadly, our results highlight the importance of ethnicity-specific microbiome studies to assess the risk of PTB, especially in otherwise low-risk women.

## Data Availability Statement

The datasets presented in this study can be found in online repositories. The names of the repository/repositories and accession number(s) can be found below: http://www.ncbi.nlm.nih.gov/bioproject/692679.

## Ethics Statement

The studies involving human participants were reviewed and approved by the Institutional Review Board (IRB) of Sidra Medicine under (IRB protocol #1705010909), by the ethics committee of the faculty of Tropical Medicine, Mahidol University, Thailand (TMEC 15-062), the University of Oxford Central University Research, UK (OxTREC: 33-15). The patients/participants provided their written informed consent to participate in this study.

## Author Contributions

SK conceived and designed the study. SK, DC, BS, AM, TK, TB, RM, and FN designed the cohort. MK, SM, PS, MS, and DE performed the experiments. TB, RM, and FN recruited and consented the study participants. MK and SK wrote the manuscript with input from co-authors. All authors contributed to the article and approved the submitted version.

## Funding

This project SDR# 400075 is financially supported by funds from Sidra Medicine to SK. Shoklo Malaria Research Unit (SMRU), Mae Sot, Thailand, is part of the Mahidol Oxford Research Unit, supported by the Wellcome Trust of Great Britain.

## Conflict of Interest

The authors declare that the research was conducted in the absence of any commercial or financial relationships that could be construed as a potential conflict of interest.

## Supplementary Material

Supplementary Figure 1 Vaginal microbial diversity analysis based on ethnicity. **a**, Beta diversity analysis to estimate the dissimilarity and similarity of bacterial communities between Burman and Karen population. Principal coordinates analysis (PCoA) derived from the dissimilarity matrix of unweighted UniFrac distance. Statistical analysis calculated using anosim analysis. **b**, Alpha diversity of the microbiome in all samples collected was measured between the two ethnic groups by the number of operational taxonomic units (OTUs) observed and by the Chao1, Shannon and Simpson diversity indices.

Supplementary Figure 2 Bacterial Nugent score during pregnancy. Stacked area charts representing the percentage of women with full term birth (TB) and (PTB) with either normal, intermediate or bacterial vaginosis (BV) Nugent scores in each trimester (T-1, T-2, and T-3) and at delivery.

Supplementary Figure 3 Longitudinal vaginal microbiome profile at phylum level during pregnancy. Each phylum is shown in a different color. Columns marked on the x-axis represent samples from individual women in the full term birth (TB, upper row) and preterm birth (PTB, lower row) cohorts; individual plots represent samples at each trimester (T-1, T-2, and T-3) and at delivery.

Supplementary Figure 4 Canonical correlation analysis of vaginal microbial signature and cytokine levels at the time of delivery. The vaginal microbial taxonomic profiles and cytokine levels in samples collected from women who experienced a, TB and b, PTB at the delivery were log-transformed and co-integrated using canonical correlation analysis. Pbuc, *Prevotella buccalis*; Fine, *Finegoldia*; Chla, *Chlamydia trachomatis*; Lcri, *Lactobacillus crispatus*; Line, *Lactobacillus iners*; Lgas, *Lactobacillus gasseri;* Ljen, *Lactobacillus jensenii;* Prev, *Prevotella* 6; *Ssan, Sneathia sanguinegens*; Upar, *Ureaplasma parvum; Sam, Sneathia amnii*.

## References

[B1] Amabebe E. Anumba D. O. C. (2018). The Vaginal Microenvironment: The Physiologic Role of Lactobacilli. Front. Med. (Lausanne) 5, 181. 10.3389/fmed.2018.00181 29951482 PMC6008313

[B2] Andrews S. (2010). FastQC: a quality control tool for high throughput sequence data. Available at: http://www.bioinformatics.babraham.ac.uk/projects/fastqc.

[B3] Beck S. Wojdyla D. Say L. Betran A. P. Merialdi M. Requejo J. H.. (2010). The worldwide incidence of preterm birth: a systematic review of maternal mortality and morbidity. Bull. World Health Organ 88, 31–38. 10.2471/BLT.08.062554 20428351 PMC2802437

[B4] Blencowe H. Cousens S. Oestergaard M. Z. Chou D. Moller A. B. Narwal R.. (2012). National, regional, and worldwide estimates of preterm birth rates in the year 2010 with time trends since 1990 for selected countries: a systematic analysis and implications. Lancet 379, 2162–2172. 10.1016/S0140-6736(12)60820-4 22682464

[B5] Bolger A. M. Lohse M. Usadel B. (2014). Trimmomatic: a flexible trimmer for Illumina sequence data. Bioinformatics (Oxford England) 30, 2114–2120. 10.1093/bioinformatics/btu170 PMC4103590 24695404

[B6] Brocklehurst P. Gordon A. Heatley E. Milan S. J. (2013). Antibiotics for treating bacterial vaginosis in pregnancy. Cochrane Database Syst. Rev. 31 (1), CD000262. 10.1002/14651858.CD000262.pub4 PMC11307253 23440777

[B7] Brown R. G. Marchesi J. R. Lee Y. S. Smith A. Lehne B. Kindinger L. M.. (2018). Vaginal dysbiosis increases risk of preterm fetal membrane rupture, neonatal sepsis and is exacerbated by erythromycin. BMC Med. 16, 9. 10.1186/s12916-017-0999-x 29361936 PMC5782380

[B8] Brummaier T. Syed Ahamed Kabeer B. Lindow S. Konje J. C. Pukrittayaamee S. Utzinger J.. (2019). A prospective cohort for the investigation of alteration in temporal transcriptional and microbiome trajectories preceding preterm birth: a study protocol. BMJ Open 9, e023417. 10.1136/bmjopen-2018-023417 PMC6340419 30782707

[B9] Brummaier T. Syed Ahamed Kabeer B. Wilaisrisak P. Pimanpanarak M. Win A. K. Pukrittayakamee S.. (2020). Cohort profile: molecular signature in pregnancy (MSP): longitudinal high-frequency sampling to characterise cross-omic trajectories in pregnancy in a resource-constrained setting. BMJ Open 10, e041631. 10.1136/bmjopen-2020-041631 PMC7549449 33040018

[B10] Callahan B. J. DiGiulio D. B. Goltsman D. S. A. Sun C. L. Costello E. K. Jeganathan P.. (2017). Replication and refinement of a vaginal microbial signature of preterm birth in two racially distinct cohorts of US women. Proc. Natl. Acad. Sci. U. S. A. 114, 9966–9971. 10.1073/pnas.1705899114 28847941 PMC5604014

[B11] Caporaso J. G. Kuczynski J. Stombaugh J. Bittinger K. Bushman F. D. Costello E. K.. (2010). QIIME allows analysis of high-throughput community sequencing data. Nat. Methods 7, 335–336. 10.1038/nmeth.f.303 20383131 PMC3156573

[B12] Chao A. (1987). Estimating the population size for capture-recapture data with unequal catchability. Biometrics 43, 783–791. 10.2307/2531532 3427163

[B13] Chawanpaiboon S. Vogel J. P. Moller A. B. Lumbiganon P. Petzold M. Hogan D.. (2019). Global, regional, and national estimates of levels of preterm birth in 2014: a systematic review and modelling analysis. Lancet Glob. Health 7, e37–e46. 10.1016/S2214-109X(18)30451-0 30389451 PMC6293055

[B14] Conlon M. A. Bird A. R. (2014). The impact of diet and lifestyle on gut microbiota and human health. Nutrients 7, 17–44. 10.3390/nu7010017 25545101 PMC4303825

[B15] Denney J. M. Nelson E. Wadhwa P. Waters T. Mathew L. Goldenberg R. L.. (2020). Cytokine profiling: variation in immune modulation with preterm birth vs. uncomplicated term birth identifies pivotal signals in pathogenesis of preterm birth. J. Perinat. Med. 9, jpm-2020–0025. 10.1515/jpm-2020-0025 PMC9849608 33035192

[B16] DiGiulio D. B. Callahan B. J. McMurdie P. J. Costello E. K. Lyell D. J. Robaczewska A.. (2015). Temporal and spatial variation of the human microbiota during pregnancy. Proc. Natl. Acad. Sci. U. S. A. 112, 11060–11065. 10.1073/pnas.1502875112 26283357 PMC4568272

[B17] Donders G. G. Bosmans E. Dekeersmaecker A. Vereecken A. Van Bulck B. Spitz B. (2000). Pathogenesis of abnormal vaginal bacterial flora. Am. J. Obstet. Gynecol. 182, 872–878. 10.1016/S0002-9378(00)70338-3 10764465

[B18] Donders G. G. Van Calsteren K. Bellen G. Reybrouck R. Van den Bosch T. Riphagen I.. (2009). Predictive value for preterm birth of abnormal vaginal flora, bacterial vaginosis and aerobic vaginitis during the first trimester of pregnancy. BJOG 116, 1315–1324. 10.1111/j.1471-0528.2009.02237.x 19538417

[B19] El Khouli R. H. Macura K. J. Barker P. B. Habba M. R. Jacobs M. A. Bluemke D. A. (2009). Relationship of temporal resolution to diagnostic performance for dynamic contrast enhanced MRI of the breast. J. Magn. Reson. Imaging 30, 999–1004. 10.1002/jmri.21947 19856413 PMC2935260

[B20] Eschenbach D. A. Hillier S. Critchlow C. Stevens C. DeRouen T. Holmes K. K. (1988). Diagnosis and clinical manifestations of bacterial vaginosis. Am. J. Obstet. Gynecol. 158, 819–828. 10.1016/0002-9378(88)90078-6 3259075

[B21] Fettweis J. M. Serrano M. G. Brooks J. P. Edwards D. J. Girerd P. H. Parikh H. I.. (2019). The vaginal microbiome and preterm birth. Nat. Med. 25, 1012–1021. 10.1038/s41591-019-0450-2 31142849 PMC6750801

[B22] Fredricks D. N. Fiedler T. L. Marrazzo J. M. (2005). Molecular identification of bacteria associated with bacterial vaginosis. N. Engl. J. Med. 353, 1899–1911. 10.1056/NEJMoa043802 16267321

[B23] Gajer P. Brotman R. M. Bai G. Sakamoto J. Schutte U. M. Zhong X.. (2012). Temporal dynamics of the human vaginal microbiota. Sci. Transl. Med. 4, 132ra152. 10.1126/scitranslmed.3003605 PMC3722878 22553250

[B24] Hong K. H. Hong S. K. Cho S. I. Ra E. Han K. H. Kang S. B.. (2016). Analysis of the Vaginal Microbiome by Next-Generation Sequencing and Evaluation of its Performance as a Clinical Diagnostic Tool in Vaginitis. Ann. Lab. Med. 36, 441–449. 10.3343/alm.2016.36.5.441 27374709 PMC4940487

[B25] Jespers V. Menten J. Smet H. Poradosu S. Abdellati S. Verhelst R.. (2012). Quantification of bacterial species of the vaginal microbiome in different groups of women, using nucleic acid amplification tests. BMC Microbiol. 12, 83. 10.1186/1471-2180-12-83 22647069 PMC3418157

[B26] Koren O. Goodrich J. K. Cullender T. C. Spor A. Laitinen K. Backhed H. K.. (2012). Host remodeling of the gut microbiome and metabolic changes during pregnancy. Cell 150, 470–480. 10.1016/j.cell.2012.07.008 22863002 PMC3505857

[B27] Koullali B. van Zijl M. D. Kazemier B. M. Oudijk M. A. Mol B. W. J. Pajkrt E.. (2020). The association between parity and spontaneous preterm birth: a population based study. BMC Pregnancy Childbirth 20, 233. 10.1186/s12884-020-02940-w 32316915 PMC7175552

[B28] Kumar M. Mathur T. Joshi V. Upadhyay D. J. Inoue S. I. Masuda N. (2018). Effect of DS-2969b, a novel GyrB inhibitor, on rat and monkey intestinal microbiota. Anaerobe 51, 120–123. 10.1016/j.anaerobe.2018.04.017 29758524

[B29] Kumar M. Singh P. Murugesan S. Vetizou M. McCulloch J. Badger J. H.. (2020). Microbiome as an Immunological Modifier. Methods Mol. Biol. 2055, 595–638. 10.1007/978-1-4939-9773-2_27 31502171 PMC8276114

[B30] Lamont R. F. (2003). Infection in the prediction and antibiotics in the prevention of spontaneous preterm labour and preterm birth. BJOG 110 Suppl 20, 71–75. 10.1046/j.1471-0528.2003.00034.x 12763116

[B31] Larsen J. M. (2017). The immune response to Prevotella bacteria in chronic inflammatory disease. Immunology 151, 363–374. 10.1111/imm.12760 28542929 PMC5506432

[B32] Ling Z. Liu X. Luo Y. Wu X. Yuan L. Tong X.. (2013). Associations between vaginal pathogenic community and bacterial vaginosis in Chinese reproductive-age women. PloS One 8, e76589. 10.1371/journal.pone.0076589 24124575 PMC3790675

[B33] Liu L. Oza S. Hogan D. Chu Y. Perin J. Zhu J.. (2016). Global, regional, and national causes of under-5 mortality in 2000-15: an updated systematic analysis with implications for the Sustainable Development Goals. Lancet 388, 3027–3035. 10.1016/S0140-6736(16)31593-8 27839855 PMC5161777

[B34] Lockwood C. J. (2002). Predicting premature delivery–no easy task. N. Engl. J. Med. 346, 282–284. 10.1056/NEJM200201243460412 11807155

[B35] Lozupone C. A. Hamady M. Kelley S. T. Knight R. (2007). Quantitative and qualitative beta diversity measures lead to different insights into factors that structure microbial communities. Appl. Environ. Microbiol. 73, 1576–1585. 10.1128/AEM.01996-06 17220268 PMC1828774

[B36] Lunde A. Melve K. K. Gjessing H. K. Skjaerven R. Irgens L. M. (2007). Genetic and environmental influences on birth weight, birth length, head circumference, and gestational age by use of population-based parent-offspring data. Am. J. Epidemiol. 165, 734–741. 10.1093/aje/kwk107 17311798

[B37] Ma Z. S. Li L. (2017). Quantifying the human vaginal community state types (CSTs) with the species specificity index. PeerJ 5, e3366. 10.7717/peerj.3366 28674641 PMC5490466

[B38] MacIntyre D. A. Chandiramani M. Lee Y. S. Kindinger L. Smith A. Angelopoulos N.. (2015). The vaginal microbiome during pregnancy and the postpartum period in a European population. Sci. Rep. 5, 8988. 10.1038/srep08988 25758319 PMC4355684

[B39] Mattei V. Murugesan S. Al Hashmi M. Mathew R. James N. Singh P.. (2019). Evaluation of Methods for the Extraction of Microbial DNA From Vaginal Swabs Used for Microbiome Studies. Front. Cell Infect. Microbiol. 9, 197. 10.3389/fcimb.2019.00197 31245304 PMC6563847

[B40] McMurdie P. J. Holmes S. (2013). phyloseq: an R package for reproducible interactive analysis and graphics of microbiome census data. PloS One 8, e61217. 10.1371/journal.pone.0061217 23630581 PMC3632530

[B41] Mwaniki M. K. Atieno M. Lawn J. E. Newton C. R. (2012). Long-term neurodevelopmental outcomes after intrauterine and neonatal insults: a systematic review. Lancet 379, 445–452. 10.1016/S0140-6736(11)61577-8 22244654 PMC3273721

[B42] Nugent R. P. Krohn M. A. Hillier S. L. (1991). Reliability of diagnosing bacterial vaginosis is improved by a standardized method of gram stain interpretation. J. Clin. Microbiol. 29, 297–301. 10.1128/JCM.29.2.297-301.1991 1706728 PMC269757

[B43] Nuriel-Ohayon M. Neuman H. Koren O. (2016). Microbial Changes during Pregnancy, Birth, and Infancy. Front. Microbiol. 7, 1031. 10.3389/fmicb.2016.01031 27471494 PMC4943946

[B44] O’Connell C. M. Ferone M. E. (2016). Chlamydia trachomatis Genital Infections. Microb. Cell 3, 390–403. 10.15698/mic2016.09.525 28357377 PMC5354567

[B45] Petricevic L. Domig K. J. Nierscher F. J. Sandhofer M. J. Fidesser M. Krondorfer I.. (2014). Characterisation of the vaginal Lactobacillus microbiota associated with preterm delivery. Sci. Rep. 4, 5136. 10.1038/srep05136 24875844 PMC4038809

[B46] Petrova M. I. Lievens E. Malik S. Imholz N. Lebeer S. (2015). Lactobacillus species as biomarkers and agents that can promote various aspects of vaginal health. Front. Physiol. 6, 81. 10.3389/fphys.2015.00081 25859220 PMC4373506

[B47] Ravel J. Gajer P. Abdo Z. Schneider G. M. Koenig S. S. McCulle S. L.. (2011). Vaginal microbiome of reproductive-age women. Proc. Natl. Acad. Sci. U. S. A. 108 Suppl 1, 4680–4687. 10.1073/pnas.1002611107 20534435 PMC3063603

[B48] Rohart F. Gautier B. Singh A. Le Cao K. A. (2017). mixOmics: An R package for ‘omics feature selection and multiple data integration. PloS Comput. Biol. 13, e1005752. 10.1371/journal.pcbi.1005752 29099853 PMC5687754

[B49] Romero R. Durum S. Dinarello C. A. Oyarzun E. Hobbins J. C. Mitchell M. D. (1989). Interleukin-1 stimulates prostaglandin biosynthesis by human amnion. Prostaglandins 37, 13–22. 10.1016/0090-6980(89)90028-2 2785698

[B50] Romero R. Dey S. K. Fisher S. J. (2014). Preterm labor: one syndrome, many causes. Science 345, 760–765. 10.1126/science.1251816 25124429 PMC4191866

[B51] Serrano M. G. Parikh H. I. Brooks J. P. Edwards D. J. Arodz T. J. Edupuganti L.. (2019). Racioethnic diversity in the dynamics of the vaginal microbiome during pregnancy. Nat. Med. 25, 1001–1011. 10.1038/s41591-019-0465-8 31142850 PMC6746180

[B52] Shah R. Mullany L. C. Darmstadt G. L. Mannan I. Rahman S. M. Talukder R. R.. (2014). Incidence and risk factors of preterm birth in a rural Bangladeshi cohort. BMC Pediatr. 14, 112. 10.1186/1471-2431-14-112 24758701 PMC4021459

[B53] Shannon C. E. (1948). A mathematical theory of communication, Part II. Bell Syst. Tech. J. 27, 623–656. 10.1002/j.1538-7305.1948.tb00917.x

[B54] Sharma S. Khan I. A. Ali I. Ali F. Kumar M. Kumar A.. (2009). Evaluation of the antimicrobial, antioxidant, and anti-inflammatory activities of hydroxychavicol for its potential use as an oral care agent. Antimicrob. Agents Chemother. 53, 216–222. 10.1128/AAC.00045-08 18573934 PMC2612173

[B55] Shipitsyna E. Roos A. Datcu R. Hallen A. Fredlund H. Jensen J. S.. (2013). Composition of the vaginal microbiota in women of reproductive age–sensitive and specific molecular diagnosis of bacterial vaginosis is possible? PloS One 8, e60670. 10.1371/journal.pone.0060670 23585843 PMC3621988

[B56] Simpson E. H. (1949). Measurement of Diversity. Nature 163, 688–688. 10.1038/163688a0

[B57] Singh P. Kumar M. Al Khodor S. (2019). Vitamin D Deficiency in the Gulf Cooperation Council: Exploring the Triad of Genetic Predisposition, the Gut Microbiome and the Immune System. Front. Immunol. 10, 1042. 10.3389/fimmu.2019.01042 31134092 PMC6524467

[B58] Soma-Pillay P. Nelson-Piercy C. Tolppanen H. Mebazaa A. (2016). Physiological changes in pregnancy. Cardiovasc. J. Afr. 27, 89–94. 10.5830/CVJA-2016-021 27213856 PMC4928162

[B59] Summerer M. Horst J. Erhart G. Weissensteiner H. Schonherr S. Pacher D.. (2014). Large-scale mitochondrial DNA analysis in Southeast Asia reveals evolutionary effects of cultural isolation in the multi-ethnic population of Myanmar. BMC Evol. Biol. 14, 17. 10.1186/1471-2148-14-17 24467713 PMC3913319

[B60] Verwijs M. C. Agaba S. K. Darby A. C. van de Wijgert J. (2020). Impact of oral metronidazole treatment on the vaginal microbiota and correlates of treatment failure. Am. J. Obstet. Gynecol. 222 (157), e151–157 e113. 10.1016/j.ajog.2019.08.008 PMC6995998 31404542

[B61] Vogel J. P. Chawanpaiboon S. Moller A. B. Watananirun K. Bonet M. Lumbiganon P. (2018). The global epidemiology of preterm birth. Best Pract. Res. Clin. Obstet. Gynaecol. 52, 3–12. 10.1016/j.bpobgyn.2018.04.003 29779863

[B62] Walther-Antonio M. R. Jeraldo P. Berg Miller M. E. Yeoman C. J. Nelson K. E. Wilson B. A.. (2014). Pregnancy’s stronghold on the vaginal microbiome. PloS One 9, e98514. 10.1371/journal.pone.0098514 24896831 PMC4045671

[B63] Witten D. M. Tibshirani R. Hastie T. (2009). A penalized matrix decomposition, with applications to sparse principal components and canonical correlation analysis. Biostatistics 10, 515–534. 10.1093/biostatistics/kxp008 19377034 PMC2697346

[B64] Workalemahu T. Grantz K. L. Grewal J. Zhang C. Louis G. M. B. Tekola-Ayele F. (2018). Genetic and Environmental Influences on Fetal Growth Vary during Sensitive Periods in Pregnancy. Sci. Rep. 8, 7274. 10.1038/s41598-018-25706-z 29740100 PMC5940684

[B65] Yockey L. J. Iwasaki A. (2018). Interferons and Proinflammatory Cytokines in Pregnancy and Fetal Development. Immunity 49, 397–412. 10.1016/j.immuni.2018.07.017 30231982 PMC6152841

[B66] Yoshimura K. Morotomi N. Fukuda K. Nakano M. Kashimura M. Hachisuga T.. (2011). Intravaginal microbial flora by the 16S rRNA gene sequencing. Am. J. Obstet. Gynecol. 205, 235 e231–e239. 10.1016/j.ajog.2011.04.018 21783170

[B67] Zeller G. Tap J. Voigt A. Y. Sunagawa S. Kultima J. R. Costea P. I.. (2014). Potential of fecal microbiota for early-stage detection of colorectal cancer. Mol. Syst. Biol. 10, 766. 10.15252/msb.20145645 25432777 PMC4299606

[B68] Zhang J. Kobert K. Flouri T. Stamatakis A. (2014). PEAR: a fast and accurate Illumina Paired-End reAd mergeR. Bioinformatics (Oxford England) 30, 614–620. 10.1093/bioinformatics/btt593 PMC3933873 24142950

