# Supplementary figures and images for: Vaginal Microbiota and Cytokine Levels Predict Preterm Delivery in Asian Women
Source: Front Cell Infect Microbiol. 2021 Mar 4;11:639665. doi: 10.3389/fcimb.2021.639665 (PMC7969986; doi:10.3389/fcimb.2021.639665)

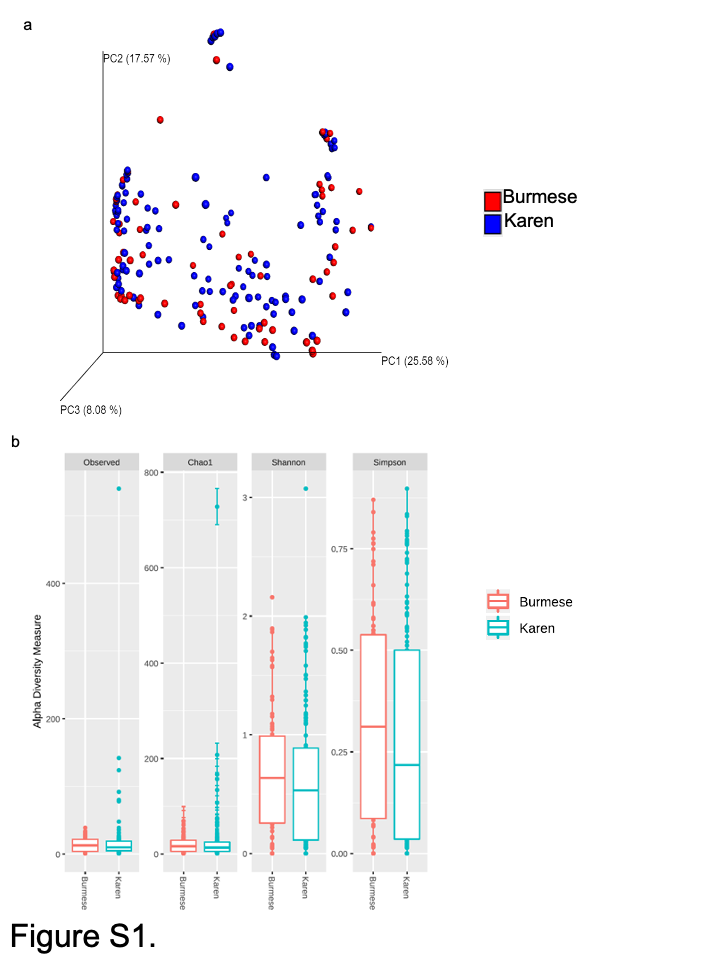

Supplement: Supplementary Figure 1 — Vaginal microbial diversity analysis based on ethnicity. a, Beta diversity analysis to estimate the dissimilarity and similarity of bacterial communities between Burman and Karen population. Principal coordinates analysis (PCoA) derived from the dissimilarity matrix of unweighted UniFrac distance. Statistical analysis calculated using anosim analysis. b, Alpha diversity of the microbiome in all samples collected was measured between the two ethnic groups by the number of operational taxonomic units (OTUs) observed and by the Chao1, Shannon and Simpson diversity indices. [file Image_1.tiff]

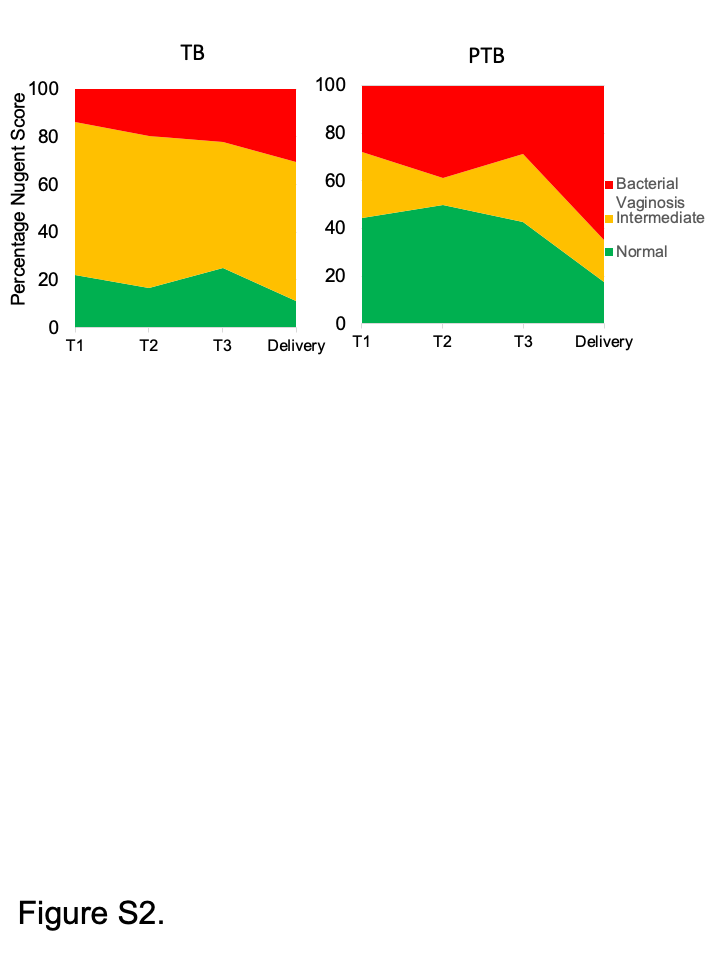

Supplement: Supplementary Figure 2 — Bacterial Nugent score during pregnancy. Stacked area charts representing the percentage of women with full term birth (TB) and (PTB) with either normal, intermediate or bacterial vaginosis (BV) Nugent scores in each trimester (T-1, T-2, and T-3) and at delivery. [file Image_2.tiff]

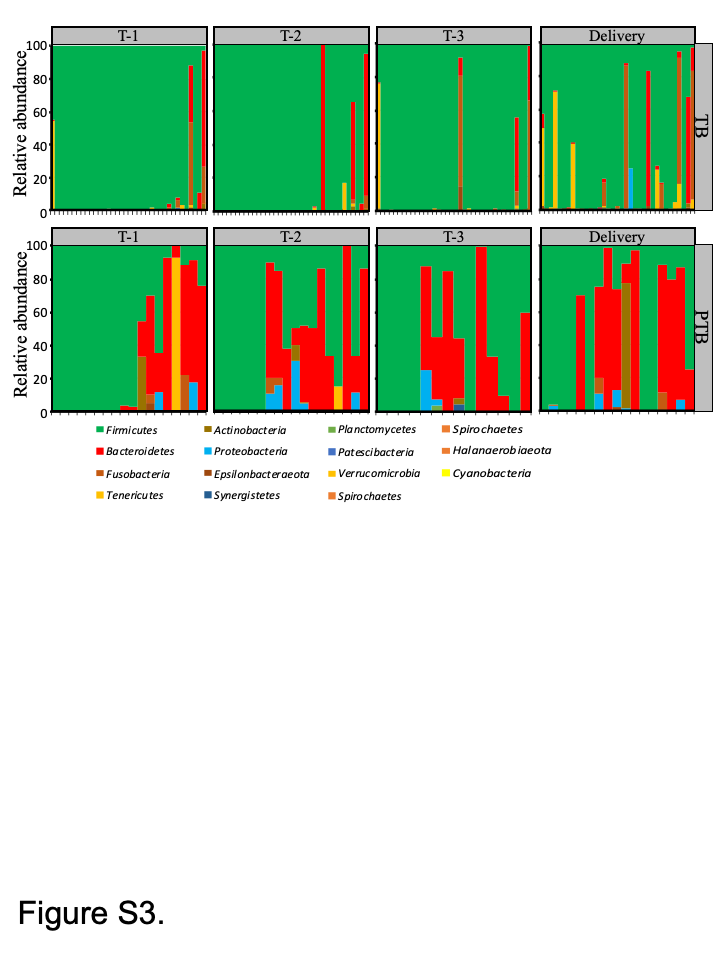

Supplement: Supplementary Figure 3 — Longitudinal vaginal microbiome profile at phylum level during pregnancy. Each phylum is shown in a different color. Columns marked on the x-axis represent samples from individual women in the full term birth (TB, upper row) and preterm birth (PTB, lower row) cohorts; individual plots represent samples at each trimester (T-1, T-2, and T-3) and at delivery. [file Image_3.tiff]

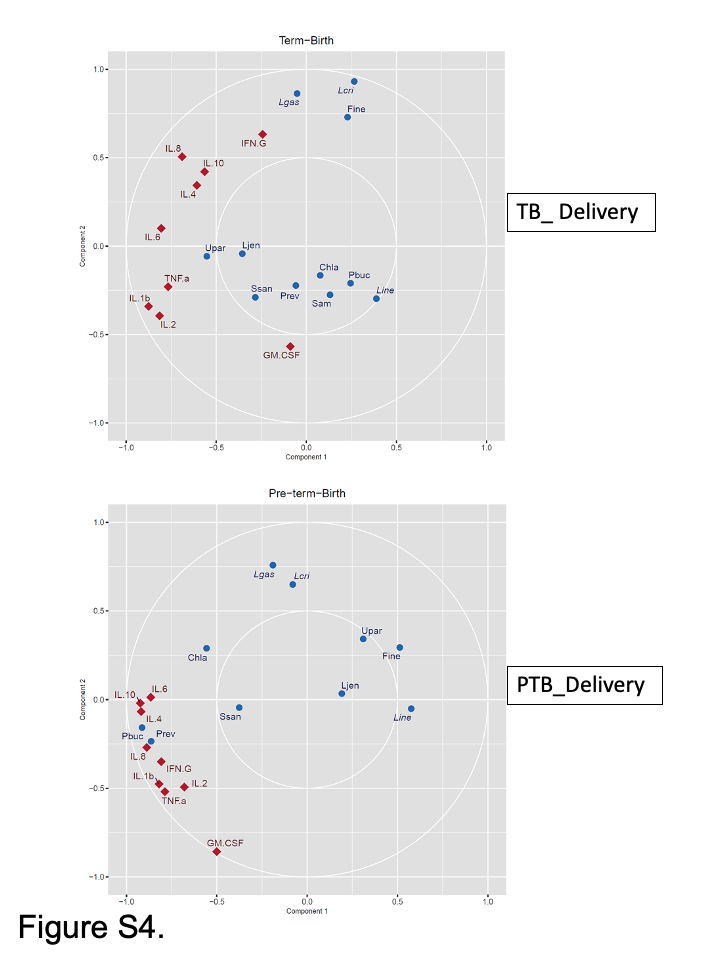

Supplement: Supplementary Figure 4 — Canonical correlation analysis of vaginal microbial signature and cytokine levels at the time of delivery. The vaginal microbial taxonomic profiles and cytokine levels in samples collected from women who experienced a, TB and b, PTB at the delivery were log-transformed and co-integrated using canonical correlation analysis. Pbuc, Prevotella buccalis; Fine, Finegoldia; Chla, Chlamydia trachomatis; Lcri, Lactobacillus crispatus; Line, Lactobacillus iners; Lgas, Lactobacillus gasseri; Ljen, Lactobacillus jensenii; Prev, Prevotella 6; Ssan, Sneathia sanguinegens; Upar, Ureaplasma parvum; Sam, Sneathia amnii. [file Image_4.tiff]
