# Supplementary material for: Vaginal Microbiota and Cytokine Levels Predict Preterm Delivery in Asian Women
Source: Front Cell Infect Microbiol. 2021 Mar 4;11:639665. doi: 10.3389/fcimb.2021.639665 (PMC7969986; doi:10.3389/fcimb.2021.639665)
Supplement: Supplementary file 5 [file Table_1.docx]

Table S1. Comparison of Term vs Preterm cases of the 1:2 Case: Control selection.

|  | TB (n= 36) | | | PTB (n=18) | P-value |
| --- | --- | --- | --- | --- | --- |
| Inclusion EGA, days; Median (range) | 81 (56 - 93) | | | 83 (60 - 94) | 0.776* |
| Maternal age; Median (IQR) | 24 (21 - 27) | | | 21.5 (20 - 24.5) | 0.265* |
| Maternal age groups, n(%) |  | | |  |  |
| - 18-24 | 20 (55.6) | | | 13 (72.2) |  |
| - 25-29 | 10 (27.8) | | | 2 (11.1) |  |
| - 30-34 | 5 (13.9) | | | 3 (16.7) |  |
| - 35-39 | 1 (2.8) | | | 0 (0.0) |  |
| - ≥40 | 0 (0.0) | | | 0 (0.0) |  |
| Ethnicity, n(%) |  | | |  |  |
| - Karen | 25 (69.4) | | | 10 (55.6) | 0.314^$^ |
| - Burman | 11 (30.6) | | | 8 (44.4) |  |
| Gravidity; Median (IQR) | 2 (1 - 3) | | | 2 (1 - 2) | 0.291* |
| Gravida groups, n(%) |  | | |  |  |
| - Primigravida | 13 (36.1) | | | 8 (44.4) |  |
| - 2 | 13 (36.1) | | | 8 (44.4) |  |
| - 3 | 5 (13.9) | | | 1 (5.6) |  |
| - 4 | 4 (11.1) | | | 1 (5.6) |  |
| - ≥5 | 1 (2.8) | | | 0 (0.0) |  |
| Parity; Median (IQR) | 1 (0 - 1) | | | 0.5 (0 - 1) | 0.250* |
| Parity groups, n(%) |  |  |  |  |  |
| - 0 (nullipara) | 14 (38.9) | | | 9 (50.0) |  |
| - 1 | 14 (38.9) | | | 8 (44.4) |  |
| - 2 | 5 (13.9) | | | 0 (0.0) |  |
| - 3 | 3 (8.3) | | | 1 (5.6) |  |
| - ≥4 | 0 (0.0) | | | 0 (0.0) |  |
| Literate, n(%) | 28 (77.8) | | | 12 (66.7) | 0.583^$^ |
| Smoking, n(%) | 0 (0.0) | | | 0 (0.0) | NA |
| Obstetric history (if gravida ≥2) |  | | |  |  |
| Miscarriage, n(%) | 5 (21.7) | | | 2 (20.0) | 1.000^‡^ |
| Stillbirth, n(%) | 0 (0.0) | | | 0 (0.0) | NA |
| PIH, n(%) | 0 (0.0) | | | 0 (0.0) | NA |
| Preterm rupture of membranes, n(%) | 0 (0.0) | | | 2 (20.0) | 0.085^‡^ |
| Preterm labour, n(%) | 1 (4.3) | | | 5 (50.0) | 0.005^‡^ |
|  |  | | |  |  |
| Enrolment examination data | Term (n= 36) | | | Preterm (n=18) | P-value |
| Weight (kg); median, IQR | 48 (44.25 - 55.25) | | | 48 (42.25 - 48.875) | 0.334* |
| Height (cm); median (IQR) | 151.6 (149.2 - 155.425) | | | 154.15 (150.475 - 155.575) | 0.267* |
| MUAC (cm); median (IQR) | 25.95 (24.075 - 28.55) | | | 25.05 (23 - 27) | 0.263* |
| Enrolment BMI; median (IQR) | 20.86 (19.3275 - 23.39) | | | 20.125 (18.185 - 20.43) | 0.150* |
| BMI groups; n(%) |  | | |  |  |
| - Underweight | 5 (13.9) | | | 5 (27.8) |  |
| - Normal weight | 21 (58.3) | | | 11 (61.1) |  |
| - Overweight | 8 (22.2) | | | 1 (5.6) |  |
| - Obese | 2 (5.6) | | | 1 (5.6) |  |
| HCT (%) at ANC enrolment; Median (IQR) | 36.5 (34 - 39) | | | 36.5 (34.25 - 38) | 0.760* |
| HepBsAg positive; n(%) | 0 (0.0) | | | 1 (5.6) | 0.333^‡^ |
| Reactive VDRL; n(%) | 0 (0.0) | | | 1 (5.6) | 0.333^‡^ |
| HIV positive; n(%) | 0 (0.0) | | | 0 (0.0) | NA |
| Gestational diabetes; n(%) | 6 (16.7) | | | 1 (5.6) | 0.403^‡^ |
| Pre-eclampsia; n(%) | 0 (0.0) | | | 1 (5.6) | 0.333^‡^ |
| Pregnancy induced hypertension; n(%) | 2 (5.6) | | | 0 (0.0) | 0.547^‡^ |
|  |  | | |  |  |
| Outcome data | Term (n= 36) | | | Preterm (n=18) | P-value |
| Outcome EGA (days); Median (IQR) | 276.5 (269.75-283) | | | 253.5 (242-254.75) | <0.001* |
| Preterm categories; n(%) |  | | |  |  |
| - Term (≥37) | 36 (100.0) | | | 0 (0.0) |  |
| - Moderate/late PTB (≥32) | 0 (0.0) | | | 16 (88.9) |  |
| - Very PTB (28 – <32) | 0 (0.0) | | | 2 (11.1) |  |
| - Extremely PTB (<28) | 0 (0.0) | | | 0 (0.0) |  |
| Infant sex (male) ; n(%) | 18 (50.0) | | | 10 (55.6) | 0.923^$^ |
| Apgar after 1 min; Median (IQR) | 9 (9-9) | | | 9 (9-9) | 0.364* |
| Apgar after 5 min; Median (IQR) | 10 (10-10) | | | 10 (10-10) | 0.587* |
| Resuscitation; n(%) |  | | |  |  |
| - Yes | 0 (0.0) | | | 1 (5.6) | 0.125^$^ |
| - No | 36 (100.0) | | | 16 (88.9) |  |
| - Unknown | 0 (0.0) | | | 1 (5.6) |  |
| Abnormal newborn exam; n(%) | 0 (0.0) | | | 1 (5.6) | 0.333^‡^ |
| Average birthweight (g) Median (IQR) | 3060 (2907.5-3310) | | | 2265 (1980-2440) | <0.001* |
| Birthweight categories ; n(%)  (some cases missing) |  | | |  |  |
| - Small for GA | 1 (2.9) | | | 4 (23.5) |  |
| - Appropriate for GA | 33 (94.3) | | | 13 (76.5) |  |
| - Large for GA | 1 (2.9) | | | 0 (0.0) |  |
| Average infant length (cm), Median (IQR) | 48.8 (48.5-49.6) | | | 45.6 (43.9-46.6) | <0.001* |
| Length; n(%)  (some cases missing) |  | | |  |  |
| - Short for GA | 2 (5.7) | | | 2 (11.8) |  |
| - Appropriate for GA | 31 (88.6) | | | 15 (88.2) |  |
| - Tall for GA | 2 (5.7) | | | 0 (0.0) |  |
| Head circumference at birth Median (IQR) | 33 (32.5-33.7) | | | 31.0 (30-31.5) | <0.001* |
| Head circumference ; n(%)  (some cases missing) |  | | |  |  |
| - Small HC for GA | 9 (25.7) | | | 4 (23.5) |  |
| - Appropriate HC for GA | 26 (74.3) | | | 13 (76.5) |  |
| - Large HC for GA | 0 (0.0) | | | 0 (0.0) | NA |
| Caesarean section; n(%) | 1 (2.8) | | | 0 (0.0) | 1^‡^ |
| Breech delivery; n(%) | 0 (0.0) | | | 1 (5.6) | 0.333^‡^ |
| Vacuum delivery; n(%) | 1 (2.8) | | | 0 (0.0) | 1^‡^ |
| Delivered by; n(%) |  | | |  |  |
| - Midwife | 34 (94.4) | | | 16 (88.9) | 0.080^$^ |
| - TBA | 0 (0.0) | | | 2 (11.1) |  |
| - Doctor | 2 (5.6) | | | 0 (0.0) |  |
| Place of delivery; n(%) |  | | |  |  |
| - SMRU clinic | 33 (91.7) | | | 15 (83.3) | 0.204^$^ |
| - Home | 1 (2.8) | | | 2 (11.1) |  |
| - Thai Hospital | 2 (5.6) | | | 0 (0.0) |  |
| - On the way to clinic | 0 (0.0) | | | 1 (5.6) |  |
| Induction of labour; n(%) | 4 (11.1) | | | 1 (5.6) | 0.655^‡^ |
| Augmentation of labour; n(%) | 5 (13.9) | | | 0 (0.0) | 0.157^‡^ |
| Postpartum haemorrhage; n(%) | 2 (5.6) | | | 0 (0.0) | 0.338^‡^ |
| Estimated blood loss (mL); Median (IQR) | 100 (100-200) | | | 100 (100-142.5) | 0.625* |
| Neonatal death; n(%) | 0 (0.0) | | | 0 (0.0) | NA |
| * Mann Whitney U Test  $ Chi-square Test  ‡ Fisher Exact test  PTB: preterm birth; TB: term birth; EGA: Estimated Gestational Age | | | | | |
